# Posttranslational Modifications in Conserved Transcription Factors: A Survey of the TALE-Homeodomain Superclass in Human and Mouse

**DOI:** 10.3389/fcell.2021.648765

**Published:** 2021-03-09

**Authors:** Marina Reichlmeir, Lena Elias, Dorothea Schulte

**Affiliations:** Institute of Neurology (Edinger Institute), University Hospital Frankfurt, Goethe University, Frankfurt, Germany

**Keywords:** homeodomain protein, PTM, protein phosphorylation, MEIS, PBX, PREP/PKNOX, TGIF, IRX

## Abstract

Transcription factors (TFs) guide effector proteins like chromatin-modifying or -remodeling enzymes to distinct sites in the genome and thereby fulfill important early steps in translating the genome’s sequence information into the production of proteins or functional RNAs. TFs of the same family are often highly conserved in evolution, raising the question of how proteins with seemingly similar structure and DNA-binding properties can exert physiologically distinct functions or respond to context-specific extracellular cues. A good example is the TALE superclass of homeodomain-containing proteins. All TALE-homeodomain proteins share a characteristic, 63-amino acid long homeodomain and bind to similar sequence motifs. Yet, they frequently fulfill non-redundant functions even in domains of co-expression and are subject to regulation by different signaling pathways. Here we provide an overview of posttranslational modifications that are associated with murine and human TALE-homeodomain proteins and discuss their possible importance for the biology of these TFs.

## Introduction

TFs recognize specific DNA sequences, often depending on DNA shape or methylation status, to control the local assembly of larger protein complexes that induce the transcriptional activation or repression of nearby genes. Transcription factors (TFs) are thus vital to determining which gene product is produced when, where, in which quantities, and in response to what external signal(s). In human, these multifaceted tasks are performed by an estimated ∼1,600 different TFs (Lambert et al., 2018). Although this seems like an impressive repertoire, TFs use a limited number of DNA binding domain (DBD) types, with most metazoan TFs belonging to the C_2_H_2_ zinc- finger-, homeodomain (HD)-, basic helix-loop-helix (bHLH)-, basic leucine zipper-, forkhead-, nuclear hormone receptor-, or high-mobility group (HMG)/SRY-related HMG-box (SOX)-superclasses. DBD-types are highly variable across classes but very similar in TFs belonging to the same class. Evolutionary related TFs often also share extensive sequence similarity outside of the DBD. This raises the conundrum how physiologically distinct functions may be carried out by proteins that possess the same overall structure and, at least *in vitro*, nearly identical DNA-binding properties.

TFs almost always function as ensembles, consistent with the concept that the composition of the multiprotein complex dictates the affinity and specificity of DNA binding (Slattery et al., 2011; Bridoux et al., 2020). The ability of a TF to interact with DNA or with other proteins depends on the biochemical properties of the amino acids involved in binding, which in turn can be profoundly altered by the attachment of additional chemical moieties in a process known as posttranslational modification (PTM). Consequently, the type of binding partners a TF assembles with, the sequence motif recognized by the complex, and the strength of interaction with this motif are sensitive to PTMs (Filtz et al., 2014; Draime et al., 2018). These are important features for any TF, because the composition of transcriptional multiprotein complexes determines the cellular and physiological context in which the TF acts, while recognition of motif variations can lead to high- or low affinity DNA binding, which in turn may result in dynamic gene expression levels (Crocker et al., 2016). In this minireview, we manually surveyed high-throughput proteomics studies, published in peer-reviewed journals or deposited to open-source platforms, to compile PTMs that were recorded in TALE-HD TFs isolated from various murine and human sources. Comparing these PTMs between paralog and ortholog proteins revealed general principles by which PTMs may shape the activity of individual members of conserved TF protein families.

## TALE-HD Proteins

**FIGURE 1:**
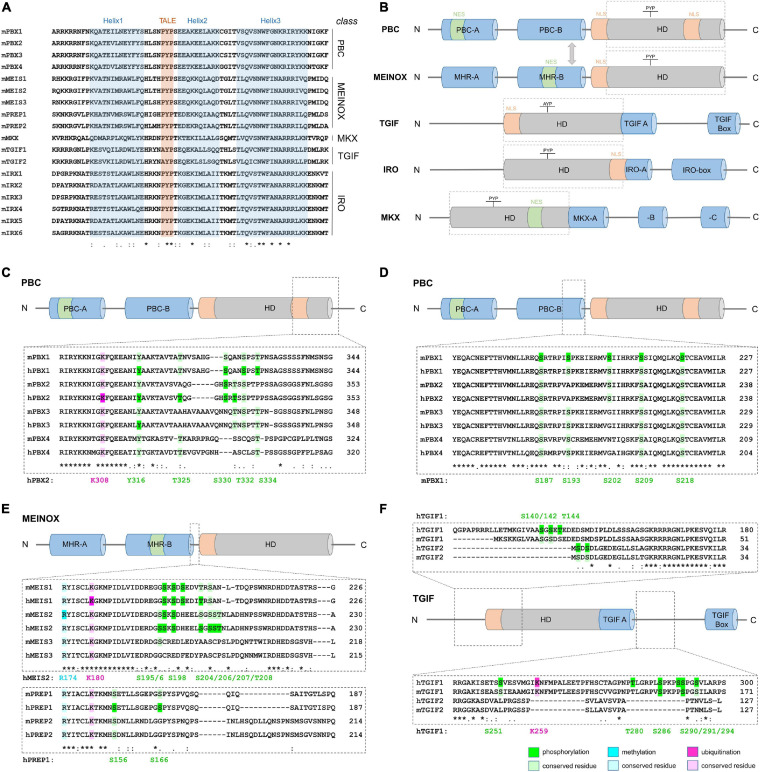
Structure of TALE-HD proteins and examples of abundant, class-specific PTMs. **(A)** Comparison of the amino acid sequence of mouse TALE-HD domains. Helices 1–3 are highlighted in blue, the name-giving TALE-motif in red. **(B)** Domain structure of the five TALE-HD protein classes. The HD is shown as gray cylinder, conserved protein domains outside the HD in blue; nuclear export signal (NES): green; nuclear localization signal (NLS): orange. See text for details. Domain sizes are not drawn to scale. **(C–E)** PTM comparison among paralogs and between human and mouse orthologs. **(C)** Lysine-ubiquitination and serine-, threonine- and tyrosine-phosphorylation C-terminal to the HD in PBC class proteins. **(D)** Serine-phosphorylation in PBC class proteins N-terminal to the HD. **(E)** Arginine-methylation, lysine-ubiquitination and serine/threonine-phosphorylation in MEINOX-proteins. **(F)** Ubiquitination and phosphorylation in TGIF-class proteins. Numeration of modified amino acids in reference proteins is shown below each amino acid alignment. Color code: green: phosphorylation, blue: methylation, pink: ubiquitination; dark shades indicate PTMs, bright shades indicate residue conservation. A list of PTMs assessed in PBC, MEIS/PREP and TGIF as well as PTMs detected in IRO and MKX can be found in Table 1.

Three amino acid loop extension-homeodomain (TALE-HD) TFs are evolutionary highly conserved and found in single-cell eukaryotes (e.g., Mata1/Mata2 in yeast), plants (e.g., KNOX and BELL), and animals (see below; Mukherjee and Bürglin, 2007). The TALE-HD differs from the canonical, 60 amino acid-long HD by the insertion of three extra residues between helix 1 and helix 2 of the HD. This motif, known as the TALE-motif, forms a hydrophobic pocket to mediate protein-protein interactions (Figure 1A; Bürglin, 1997; Piper et al., 1999; LaRonde-LeBlanc and Wolberger, 2003; Mukherjee and Bürglin, 2007). For this feature, TALE-HD proteins have been classified as “atypical” HD proteins. In animals, they have been grouped into five classes, PBC, MEINOX, TGIF, IRO and MKX, based on the sequence of the HD itself and conserved, class-specific motifs flanking the HD (Figure 1B). The developmental functions of individual TALE-HD genes and the defects associated with their mutation in animal models or in human diseases have been covered by a series of excellent recent reviews and will therefore not be discussed in detail (Kim et al., 2012; Blasi et al., 2017; Schulte and Geerts, 2019; Selleri et al., 2019). Instead, we here provide an overview of the different PTMs detected in mouse and human TALE-HD TFs and explore how such PTMs may help to convey functional specificity among these structurally similar proteins.

### PBC-Class

Mammals have four *Pbx* (pre-B-cell leukemia homeobox) genes: *Pbx1*, which was first identified in acute pre-B-cell leukemias, and *Pbx2* to *Pbx4*, which were identified later by sequence homology to *Pbx1* (Figure 1A; Kamps et al., 1990; Wagner et al., 2001; Rhee et al., 2004; Selleri et al., 2004). PBC-class TFs dimerize with MEINOX-class proteins through a conserved 180-amino acid-long domain termed PBC-homology domain (Figure 1B; Bürglin and Ruvkun, 1992; Bruckmann et al., 2020). Monomeric PBX proteins or PBX-MEINOX dimers form cell type-specific transcriptional regulatory units with other TFs, including HD transcription factor like HOX-proteins, basic helix-loop-helix (bHLH), nuclear receptors, Smad2,3,4 intracellular signaling proteins of the TGF-β pathway, as well as chromatin modifying enzymes (Peltenburg and Murre, 1996; Wang et al., 2001; Subramaniam et al., 2003; Bailey et al., 2004; Choe et al., 2009, 2014; Merabet and Galliot, 2015). In fact, PBC-class proteins are essential co-factors of HOX-proteins, which themselves are subject to multiple forms of PTM (Draime et al., 2018). *Pbx1*, *Pbx2*, and *Pbx3* are extensively co-expressed and can partly compensate for each other in domains of co-expression (Selleri et al., 2001, 2004; Rhee et al., 2004; Capellini et al., 2006). Genetic mutant models in rodents are diverse, but defective skeletal patterning and hematopoiesis, as well as hypoplasia and defective development of multiple organs, including pancreas, spleen, face, heart, vascular system, and brain are frequent phenotypes (Selleri et al., 2019).

### MEINOX-Class

The vertebrate MEINOX-class is divided into two subclasses, MEIS (Myeloid ecotropic viral integration site) with three genes in mammals, *Meis1-3*, and PREP/PKNOX (Pbx-regulating protein/PBX-Knotted Homeobox) with *Prep1 and Prep2*. *Meis* and *Prep* are widely co-expressed, compete for heterodimerization with PBC-class proteins and play opposing roles in tumorigenesis (Dardaei et al., 2014). *MEIS1* is upregulated in many human cancers, including the majority of acute myeloid leukemias (AML), whereas *PREP1* has tumor-suppressive properties (Lawrence et al., 1999; Longobardi et al., 2010; Blasi et al., 2017; Schulte and Geerts, 2019). In addition to the C-terminal HD, MEINOX proteins possess a N-terminal bipartite domain, termed MEIS homology region (MHR) -A and -B, which mediates the binding to their PBX partners (Figure 1B; Bürglin, 1997; Knoepfler et al., 1997; Shanmugam et al., 1999; Bruckmann et al., 2020). Mutant mouse models exist mostly for *Meis1* and *Prep1*. Consistent with MEIS’ or PREP’s heterodimerization with PBX1, many defects associated with *Meis1* or *Prep1* loss-of-function overlap with those seen in *Pbx1* mutants.

### More Distantly Related TALE-HD Proteins: TGIF-, IRO- and MKX-Classes

*Tgif1* and *Tgif2* (Transforming growth factor beta (TGF-β)-induced factor/TG-interacting factor) are phylogenetically most closely related to the MEINOX class (Mukherjee and Bürglin, 2007). They carry a distinct variation of the TALE-motif, AYP, instead of the PYP found in all other TALE-HD proteins (Figure 1A) as well as two short sequence motifs C-terminal to the HD (Figure 1B). TGIF proteins are transcriptional repressors that have been implicated in the regulation of various signaling pathways, most prominently TGF-β- and retinoic acid signaling (Bertolino et al., 1995; Wotton et al., 1999; Shen and Walsh, 2005; Guca et al., 2018). Loss-of-function phenotypes for *Tgif1* in mice are strain-dependent and range from no overt defect to holoprosencephaly, a brain malformation that has also been linked to *TGIF1* mutations in humans (Kuang et al., 2006; Taniguchi et al., 2012). Constituting another TALE-HD class, the six mammalian *Irx* genes, taking their names from the *Iroquois* complex in *D. melanogaster*, are located in two paralogous clusters in the genome and characterized by a bipartite IRO-box C-terminal of the HD (Figure 1B; Peters et al., 2000; Mukherjee and Bürglin, 2007). Loss-of-function models in mice were generated for all six *Irx* genes and established that *Irx3*, *-4* and *-5* are important transcriptional regulators in the developing and adult heart, that *Irx1* controls lung- and tooth development, and that *Irx5*- and *-6* participate in retina development (Bruneau et al., 2001; Costantini et al., 2005; Zhang et al., 2011; Gaborit et al., 2012; Star et al., 2012; Yu et al., 2017). Finally, the single gene Mohawk (*Mkx*, also known as iroquois homeobox protein-like 1) most closely related to IRX but recognized as separate class, plays a prominent role in tendon development (Mukherjee and Bürglin, 2007; Ito et al., 2010).

In short, members of the same class of TALE-HD proteins share a high degree of sequence similarity, are frequently co-expressed, and functionally cooperate in some physiological contexts but fulfill unique developmental functions in others.

## PTMs in TALE-HD Proteins

We manually surveyed 26 high-resolution and/or quantitative mass-spectrometry analyses, as well as data deposited in the open-source platform PhosphoSitePlus^®^ to compile PTMs that had been detected in mouse or human TALE-HD proteins (Table 1). Although this information is freely available in the supporting information of the respective publications, it had not been systematically assessed nor had the data been compared among studies or between protein groups. We limited our search to the three PTMs that were most frequently detected in these studies: phosphorylation, lysine-ubiquitination and arginine-methylation. This search identified a total of 187 distinct phosphorylation sites, 11 ubiquitinated and 3 methylated residues. Many of these PTMs were detected in various physiological contexts and across species, suggesting that common regulatory mechanisms apply. Particularly arginine-methylation and lysine-ubiquitination occurred almost exclusively at amino acids that were highly conserved among paralogs, indicating that significant evolutionary pressure may act on these residues (Figures 1C,E,F). The amino acid arginine forms more hydrogen bonds with protein or DNA than any other amino acid, with particularly strong bonds formed with guanine bases and the DNA phosphate backbone (Luscombe et al., 2001). Arginine residues are therefore important to stabilize the intra- and intermolecular interaction of amino acids in proteins and multiprotein complexes as well as the contact of proteins to DNA (Luscombe et al., 2001; Bedford and Clarke, 2009; Lorton and Shechter, 2019). Consequently, methylation of arginine residues in TFs can profoundly alter their function. In fact, although the significance of arginine-methylation in hPBX2 and hMEIS1 is still unknown, methylation of R174 in mMEIS2 controls nucleo-cytoplasmic translocation (Kolb et al., 2018).

**TABLE 1:** Summary of post-translational modifications of TALE-HD proteins.

Species	Protein	Motifs*	PTMs**	Detection***	References****
**PBC class**					
Human	PBX1	PBC-A: 43–122 PBC-B: 140–232 HD: 236–298	**Phosphorylation:** *S126*, S136, S141, S144, *Y305*, *S321*, S325, T328	qMS; HeLa cells MS; HeLa cells	Kettenbach et al., 2011 Sharma et al., 2014 *PhosphoSitePlus*
			**Ubiquitination:** K87, K195	MS; HEP2, Jurkat cells	Akimov et al., 2018

	PBX2	PBC-A: 53–132 PBC-B: 151–243 HD: 247–309	**Phosphorylation:** S41, S101,	qMS; HeLa, K562 cells	Pan et al., 2009
			S104, S105, S136, **S151**, S155,	qMS; hESCs	Rigbolt et al., 2011
			S159, Y316, T325, **S330**, T332,	qMS; KG1 AML cells	Weber et al., 2012
			**S395**, S423, S426, S429	qMS; U2OS cells	Beli et al., 2012
				MS; HeLa, K562 cells	Zhou et al., 2013
				qMS; CCR tumors, normal tissue; HCT116, SW480, SW620 cells	Shiromizu et al., 2013
				MS; human liver	Bian et al., 2014
				MS; HeLa cells	Sharma et al., 2014
				qMS; WM239A cells	Stuart et al., 2015
				qMS; breast tumors	Mertins et al., 2016
				qMS; HEK293 cells	Boeing et al., 2016 *PhosphoSitePlus*
			**Ubiquitination:** K97, K164, K308	qMS; HEK293 cells	Boeing et al., 2016
				MS; HEP2, Jurkat cells	Akimov et al., 2018
			**Methylation:** R15	MS; HEK293, HeLa, U2OS cells	Larsen et al., 2016 *PhosphoSitePlus*

	PBX3	PBC-A: 46–125	**Phosphorylation:** S121, Y307	MS; HeLa cells	Imami et al., 2008
		PBC-B: 134–234 HD: 238–300		MS; HeLa cells	Sharma et al., 2014 *PhosphoSitePlus*

	PBX4	PBC-A: 19–98 PBC-B: 117–209 HD: 213–275	**Phosphorylation:** S10, S33, *T153*, *S255*, *S258*	qMS; CCR tumors, normal tissue; HCT116, SW480, SW620 cells	Shiromizu et al., 2013 Mertins et al., 2016 *PhosphoSitePlus*
				qMS; breast tumors	

Mouse	PBX1	PBC-A: 43–122 PBC-B: 140–232 HD: 236–298	**Phosphorylation:** S187, S193, S202, S209, S218	2D-SDS PAGE, *in vitro* phosphorylation, site directed mutation; NIH3T3 cells	Kilstrup-Nielsen et al., 2003

	PBX2	PBC-A: 53–132 PBC–B: 151–243 HD: 247–309	**Phosphorylation:** *S136*, **S151**, S159, **S330**, **S395**, T428, S429	MS; 3 weeks old male mice	Huttlin et al., 2010
				MS; murine leukemia cell lines	Trost et al., 2012
				MS; 3T3-L1 adipocytes	Minard et al., 2016
				MS; pancreatic islet cells	Sacco et al., 2016 *PhosphoSitePlus*

**MEIS class**					
Human	MEIS1	MHD-A: 72–111 MHD-B: 136–180 HD: 275–337	**Phosphorylation:** S194, **S196**, **S198**, T202	qMS; HeLa cells qMS; KG1 AML cells	Kettenbach et al., 2011 Weber et al., 2012
				qMS; U2OS cells	Beli et al., 2012
				qMS; CCR tumors, normal tissue; HCT116, SW480, SW620 cells	Shiromizu et al., 2013
				MS; HeLa, K562 cells	Zhou et al., 2013
				MS; HeLa cells	Sharma et al., 2014
				qMS; WM239A cells	Stuart et al., 2015
				qMS; breast tumors	Mertins et al., 2016*PhosphoSitePlus*
			**Ubiquitination**: K178	MS; HEP2, Jurkat cells	Akimov et al., 2018
			**Methylation**: R383 (isoform EAW99896.1 only)	MS; HEK293, HeLa, U2OS cells	Larsen et al., 2016

	MEIS2	MHD-A: 74–113 MHD-B: 138–182 HD: 279–341	**Phosphorylation:** S195, **S196**, **S198**, S204, S206, S207, T208	qMS; HeLa, K562 cells	Pan et al., 2009
				MS; HeLa, K562 cells	Zhou et al., 2013
				qMS; WM239A cells	Stuart et al., 2015
				qMS; breast tumors	Mertins et al., 2016 *PhosphoSitePlus*
			**Ubiquitination**: K180	MS; HEP2, Jurkat cells	Akimov et al., 2018

	MEIS3	MHD-A: 54–99 MHD-B: 124–168 HD: 265–327	**Phosphorylation:** *S118*, *S124*		*PhosphoSitePlus*

**Mouse**	MEIS1	MHD-A: 72–111 MHD-B: 136–180 HD: 275–337	**Phosphorylation:** *S21*, *T22*, *Y24*, S194, **S196**, **S198**	MS; 3 weeks old male mice	Huttlin et al., 2010
				MS; murine leukemia cell lines	Trost et al., 2012
				qMS; E16.5, P0, P21 mouse brain	Doubleday and Ballif, 2014
				MS; 3T3-L1 adipocytes	Minard et al., 2016
				MS; pancreatic islet cells	Sacco et al., 2016 *PhosphoSitePlus*

	MEIS2	MHD-A: 74–113 MHD-B: 138–182 HD: 279–341	**Phosphorylation:** S196, **S198**, *S261*, *T264*	MS; 3T3-L1 adipocytes	Minard et al., 2016 *PhosphoSitePlus*
			**Methylation:** R174	MS; adult SVZ stem-/progenitor cells	Kolb et al., 2018
**PREP/PKNOX class**					
Human	PREP1	MHD-A: 52–83	**Phosphorylation:** S33, S41,	MS; HeLa cells	Kettenbach et al., 2011
		MHD-B: 109–153	*S156*, *S166*, *S239*, S324,	MS; human liver	Bian et al., 2014
		HD: 262–320	S325, S327, T329, T332	MS; HeLa cells	Sharma et al., 2014
				qMS; breast tumors	Mertins et al., 2016
				qMS; HEK293 cells	Boeing et al., 2016 *PhosphoSitePlus*
			**Ubiquitination:** K140	qMS; HEK293 cells	Boeing et al., 2016

**Mouse**	PREP1	MHD-A: 52–83 MHD-B: 109–153 HD: 262–324	**Phosphorylation:** S33, S41, S47	MS; 3 weeks old male mice	Huttlin et al., 2010 *PhosphoSitePlus*

	PREP2	MHD-A: 68–99 MHD-B: 125–169 HD: 291–353	**Phosphorylation:** S125	MS; pancreatic islet cells	Sacco et al., 2016

**TGIF class**					
Human	TGIF1	HD: 164–226 (isoform 401 aa)	**Phosphorylation:** S95, *S115*, *S117*, S140, S142, T144, S251, T280, **S286,** S290, **S291**, S294, T364, T368	*In vitro* phosphorylation, site directed mutagenesis; L-17 mink lung epithelial cells, COS-1 cells	Lo et al., 2001
				MS; H1 hESCs	Brill et al., 2009
				qMS; hESCs	Rigbolt et al., 2011
				qMS; U2OS cells	Beli et al., 2012
				MS; HeLa cells	Sharma et al., 2014
				qMS; CCR tumors, normal tissue; HCT116, SW480, SW620 cells	Shiromizu et al., 2013
				qMS; WM239A cells	Stuart et al., 2015
				qMS; breast tumors	Mertins et al., 2016 *PhosphoSitePlus*
			**Ubiquitination:** K259, K232	*In vitro* ubiquitination; MDCK, 293 cells	Ettahar et al., 2013
				MS; HEP2, Jurkat cells	Akimov et al., 2018 *PhosphoSitePlus*

	TGIF2	HD: 16–78	**Phosphorylation: S2, S4,** S109, S110, S112, S153, S159, S174, T182, T186, *T227*	qMS; HeLa, K562 cells	Pan et al., 2009
				MS; HeLa cells	Kettenbach et al., 2011
				qMS; hESCs	Rigbolt et al., 2011
				qMS; U2OS cells	Beli et al., 2012
				qMS; KG1 AML cells	Weber et al., 2012
				MS; HeLa cells	Sharma et al., 2014
				MS; HeLa, K562 cells	Zhou et al., 2013
				qMS; breast tumors	Mertins et al., 2016 *PhosphoSitePlus*
			**Ubiquitination**: K86	MS; HEP2, Jurkat cells	Akimov et al., 2018

Mouse	TGIF1	HD: 35–97 (isoform b, 272aa)	**Phosphorylation:** S13, S15, *S157*, *S162*	MS; 3 weeks old male mice	Huttlin et al., 2010 *PhosphoSitePlus*

**IRX class**					
Human	IRX1	HD: 127–189	**Phosphorylation:** T210, S267, S280, S298, S325, S433, S447	qMS; breast tumors	Mertins et al., 2016 *PhosphoSitePlus*

	IRX2	HD: 114–176	**Phosphorylation:** S186, T213, *S231*, *S233*, *S236*, S252, S254, S285, T310, T316, S317, *S325*, *S338*, S445	qMS; hESCs	Rigbolt et al., 2011
				qMS; CCR tumors, normal tissue; HCT116, SW480, SW620 cells	Shiromizu et al., 2013
				qMS; breast tumors	Mertins et al., 2016 *PhosphoSitePlus*

	IRX3	HD: 127–189	**Phosphorylation:** *S2*, S208, *S286*, S358, S365, S372, S381, *S496*, *S499*	qMS; breast tumors	Mertins et al., 2016 *PhosphoSitePlus*

	IRX4	HD: 142–204	**Phosphorylation:** *T153*, *T154*, S258, S413, S430, S473	MS; HeLa cells	Sharma et al., 2014 *PhosphoSitePlus*

	IRX5	HD: 113–175	**Phosphorylation:** *Y3*, *Y7*, *Y9*, *Y23*, *T25*, T180, S185, T237, S246, S248, **S274,** S319, S357, *S374*, S377, S383, S385, **S464**	MS; HeLa cells	Kettenbach et al., 2011
				MS; HeLa, K562 cells	Zhou et al., 2013
				MS; HeLa cells	Sharma et al., 2014
				qMS; CCR tumors, normal tissue; HCT116, SW480, SW620 cells	Shiromizu et al., 2013
				qMS; WM239A cells	Stuart et al., 2015
				qMS; breast tumors	Mertins et al., 2016*PhosphoSitePlus*

	IRX6	HD: 146–208	**Phosphorylation:** *Y139*, *S145*, S393	qMS; CCR tumors, normal tissue; HCT116, SW480, SW620 cells	Shiromizu et al., 2013 *PhosphoSitePlus*

Mouse	IRX1	HD: 127–189	**Phosphorylation:** S241, *S267*, S280, *S298*, *S447*	MS, 3 weeks old male mice	Huttlin et al., 2010 *PhosphoSitePlus*

	IRX2	HD: 115–177	**Phosphorylation:** S187	MS; 3T3-L1 adipocytes	Minard et al., 2016

	IRX3	HD: 130–192	**Phosphorylation:** S326, S329	MS, 3 weeks old male mice	Huttlin et al., 2010

	IRX5	HD: 112–174	**Phosphorylation:** *S184*, *S236*, *S465*		*PhosphoSitePlus*
**MKX class**					
Human	MKX	HD: 71–133	**Phosphorylation:** S36, *S138*, *Y146*, *T239*, S253, *Y277*, S286	qMS; CCR tumors, normal tissue; HCT116, SW480, SW620 cells	Shiromizu et al., 2013
				qMS; breast tumors	Mertins et al., 2016 *PhosphoSitePlus*

Mouse	MKX	HD: 71–133	**Phosphorylation:** *S257*		*PhosphoSitePlus*

*Conserved sequence motifs of biological significance annotated to the canonical isoforms, provided by UniProt. HD, Homeodomain; MHD, MEINOX homology domain; PBC, PBC homology domain. **Information about post-translational modifications were summarized from the references listed and/or UniProt, PhosphoSitePlus; PTMs are annotated to the isoforms as identified by UniProt ID. Residues given in italics were identified in the PhosphoSitePlus database only, references shown in bold were detected in the majority of studies referenced. ***Detection method used, cell type analyzed. CCR, colorectal cancer; E, embryonic day; hESCs, human embryonic stem cell line; qMS, quantitative mass spectrometry: iTRAQ (isobaric Tags for Relative and Absolute Quantitation), SILAC (stable isotope labeling by amino acids in cell culture) or stable-isotope dimethyl labeling mass spectrometry; MS, mass spectrometry; P, postnatal day. ****PhosphoSitePlus: www.phosphosite.org. Residues were numbered according to the following sequences (NCBI accession No): hPBX1: NP_002576; mPBX1: NP_899198.1; hPBX2: NP_002577; mPBX2: NP_059491; hPBX3: NP_006186; hPBX4: NP_079521; hMEIS1: NP_002389 and EAW99896.1; mMEIS1: NP_001180200.1; hMEIS2: NP_733777; mMEIS2: AAC529481; hMEIS3: NP_064545.1; hPREP1: NP_004562.2; mPREP1: NP_057879.2; mPREP2: XP_006510190.1; hTGIF1: AAH31268.1; mTGIF1: NP_033398.2; hTGIF2: NP_068581.1; mTGIF2: NP_775572.1; hIRX1: NP_077313.3; mIRX1: AAF63954.1; hIRX2: NP_150366.1; mIRX2: NP_034704.1; hIRX3: NP_077312.2; hIRX4: NP_057442.1; hIRX5: NP_005844.4; mIRX5: NP_061296.1; hIRX6: NP_077311.2; hMKX: NP_001229631.1; mMKX: AAI37729.1.

In ubiquitination, the 76-amino acid protein ubiquitin is covalently attached to lysine residues of protein substrates. Ubiquitination generates conjugates that widely differ in structure, size, composition, and function (Pickart, 2001). The many ways by which lysine-ubiquitination impacts on gene expression include modification of histone tails and the subsequent change in chromatin structure and the ubiquitin-guided partial processing or full degradation of TFs (Rape, 2018). The presence of several, highly conserved ubiquitination sites in TALE-HD proteins argues for important regulatory roles, although it is presently unexplored what type(s) of ubiquitin modification TALE-HD proteins carry (e.g., monomeric, polymeric, linear, branched, carrying additional PTMs or not), whether ubiquitin-conjugation targets TALE-HD proteins for degradation, and what the cellular consequences of TALE-HD protein ubiquitination are.

Compared to arginine-methylation and lysine-ubiquitination, protein phosphorylation emerges as more wide-spread and diverse type of PTM in TALE-HD proteins. Protein phosphorylation, the covalent attachment of phosphate groups on serine, threonine, or tyrosine residues, acts within milliseconds to seconds to control protein function by primarily two mechanisms: it locally changes the electrochemical properties of a protein and by this its conformation, and it creates docking sites for intermolecular protein interactions, which in turn can propagate cellular signals or create recognition sites for other post-translationally modifying enzymes that catalyze the deposition of further PTMs nearby (Filtz et al., 2014). Phosphorylation of TFs can thereby increase or decrease protein stability, control nuclear import or export, alter the secondary structure of the TF to expose or hide its DBD, and modify the DBD’s affinity to distinct sequences in the DNA resulting in high-affinity or low-affinity binding (Filtz et al., 2014). In TALE-HD proteins, phosphosites often cluster together, frequently in regions anterior or posterior of the HD (Figures 1C–F). For instance, several studies identified phosphorylated serine, threonine, and tyrosine residues in PBX family proteins just C-terminal to the TALE HD (Figure 1C). In particular phosphorylation at T325 and S330 (numeration according to hPBX2, NCBI# NP_002577) had been detected in different mouse tissues (Huttlin et al., 2010), murine pancreatic cells following glucose exposure (Sacco et al., 2016), EGF-stimulated HeLa cells (Pan et al., 2009; Sharma et al., 2014), FGF-stimulated adipocytes (Minard et al., 2016), mouse AML models and human AML cell lines (Trost et al., 2012; Weber et al., 2012), breast cancer samples (Mertins et al., 2016), human embryonic stem cells during differentiation (Rigbolt et al., 2011), and etoposide-treated human osteosarcoma (U2OS) cells during DNA damage response (Beli et al., 2012). Interestingly, only some of these residues are conserved among paralogs. For instance, while phosphorylation is frequent at S330 in PBX2 and at the corresponding S321 in PBX1, PBX3 carries an asparagine residue and PBX4 bears a microdeletion at this position, suggesting that PBX3 and PBX4 may be insensitive to the kinase networks that impact on S321/S330 in PBX1 and PBX2, respectively (Figure 1C). In addition, these differentially phosphorylated sites are close to a NLS (KRIRYKKNI; Saleh et al., 2000). Given that controlled nuclear import is an important mechanism by which the transcriptional activity of TALE-HD proteins is regulated, these observations raise the intriguing possibility that differential phosphorylation at these residues may influence nuclear localization (Mann and Abu-Shaar, 1996; Abu-Shaar et al., 1999; Berthelsen et al., 1999; Huang et al., 2003; Kolb et al., 2018). Supporting this view, protein kinase A (PKA)-mediated phosphorylation of mammalian PBX1 at S187, S193, S202, S209, and S218, all located near a second NLS (RRKRR, N-terminal to helix 1 of the HD), affect nuclear export of PBX1 (Figure 1D; Saleh et al., 2000; Kilstrup-Nielsen et al., 2003).

MEIS1 and MEIS2 proteins exhibit a striking accumulation of phosphosites clustered between the MHR-B domain and the TALE-HD, with frequent phosphorylation at serines 195, 196, 198, 204, 206, 207, and threonine 208 (numeration according to hMEIS2, NCBI# NP_733777; Figure 1E). In fact, phosphorylation at S195/S196/S198 was detected in virtually all phosphoproteomic studies that were examined for this minireview (Table 1). Nestled between these phosphosites are several aspartate and glutamate residues, amino acids with electrically changed, acidic side chains. Phosphorylation at these serine or threonine residues is therefore expected to create a strong, focal negative charge in this region of the MEIS1 and MEIS2 polypeptide.

## PTMs, a Way to Generate Functional Diversity?

Although the physiological relevance of these phosphorylation events and the signaling pathways that induce them remain to be elucidated, it is worth pointing out that none of these phosphosites are conserved in MEIS3, PREP1, or PREP2 (Figure 1E). Similarly, most of the phosphorylated amino acids that were detected in TGIF1 are not conserved in TGIF2, and vice versa (Figure 1F). Whether or not TALE-HD paralogous proteins are subject to regulation by shared kinase pathways thus appears to be dictated by the substitution of few key residues. It should be pointed out, however, that phosphorylation is a dynamic process in which phosphorylation and dephosphorylation may alternate in rather rapid cycles (Gelens and Saurin, 2018). Phosphoproteomic data hence only reflect a snapshot of a transient phosphorylation state. Lack of evidence in literature for a specific phosphorylation event can thus very well just reflect the inability of detection at a specific moment and in that specific cellular context.

Taken together, we here compiled a broad collection of PTMs in TALE-HD proteins that had been identified in unbiased, high-resolution mass-spectrometry analyses (Table 1). Few of these PTMs have been assigned a physiological function. Yet, by taking the evolutionary conservation of modification sites into account we identified both class-specific and paralog-specific PTMs. From comparing these, concepts emerge about how the combinatorial use of such PTMs may generate functional diversity from evolutionarily conserved protein structures. Specifically, we propose that the vast repertoire of PTMs, shared or not, in paralogous and orthologous TALE-HD proteins, forms the structural backbone by which individual proteins can acquire the ability to respond to context-specific extracellular signals and exert physiologically diverse functions. Although explored here only by the example of the TALE-HD superclass, similar principles may very well also apply to other evolutionarily conserved TFs. Assays based on mutational approaches now need to be developed to test these PTMs alone and in combination for their functionality and physiological relevance. Ultimately, such information can pave the way for future studies, help unravel disease processes and facilitate rational drug design.

## Author Contributions

MR, LE, and DS jointly developed and wrote the review. All authors contributed to the article and approved the submitted version.

## Conflict of Interest

The authors declare that the research was conducted in the absence of any commercial or financial relationships that could be construed as a potential conflict of interest.
